# Effect of shield placement for transient voltage mitigation due to switching surges in a 33/11 kV transformer windings

**DOI:** 10.1371/journal.pone.0240368

**Published:** 2020-10-09

**Authors:** Avinash Srikanta Murthy, Norhafiz Azis, Jasronita Jasni, Mohammad Lutfi Othman, Mohd Fairouz Mohd Yousof, Mohd Aizam Talib

**Affiliations:** 1 Advanced Lightning, Power and Energy Research Centre (ALPER), Department of Electrical and Electronic Engineering, Faculty of Engineering, Universiti Putra Malaysia, Serdang, Selangor, Malaysia; 2 Institute of Advanced Technology (ITMA), Universiti Putra Malaysia, Serdang, Selangor, Malaysia; 3 Faculty of Electrical and Electronic Engineering, Universiti Tun Hussein Onn Malaysia, Parit Raja, Johor, Malaysia; 4 TNB Research Sdn. Bhd, Kawasan Institut Penyelidikan, Kajang, Selangor, Malaysia; Northwestern Polytechnical University, CHINA

## Abstract

This study presents an investigation on the effect of shield placement for mitigation of transient voltage in a 33/11 kV, 30 MVA transformer due to Standard Switching Impulse (SSI) and Oscillating Switching Impulse (OSI) surges. Generally, the winding and insulation in transformers could experience severe voltage stress due to the external impulses i.e. switching events. Hence, it is important to examine the voltage stress and identify the mitigation action i.e. shield placements in order to reduce the adverse effect to the transformer windings. First, the resistances, inductances, and capacitances (RLC) were calculated for disc type transformer in order to develop the winding RLC equivalent circuit. The SSI and OSI transient voltage waveforms were applied to the High Voltage (HV) winding whereby the transient voltages were simulated for each disc. The resulting voltage stresses were mitigated through different configurations of electrostatic shield placements. The resonant oscillations generated due to switching surges were analysed through initial voltage distribution. The analyses on the transient voltages of the transformer winding and standard error of the slope (SEb) reveal that the location of shield placement has a significant effect on the resonant switching voltages. The increment of the shield number in the windings does not guarantee optimize mitigation of the resonant switching transient voltages. It is found that the voltage stress along the windings is linear once a floating shield is placed between the HV and Low Voltage (LV) windings of the disc-type transformer under the SSI and OSI waveforms. These findings could assist the manufacturers with appropriate technical basis for mitigation of the transformer winding against the external transient switching overvoltage surges.

## Introduction

The power system network could be subjected to transient voltage surges originating from high-frequency electromagnetic interactions between its components. Events such as lightning or switching operations could induce transients into the power system network and subsequently affect the performances of transformers [1, 2]. Switching operations in the substations during making and breaking of power lines connected to transformers could induce high oscillatory and non-linear surges [3]. Furthermore, the operation of circuit breakers can generate multiple, Very Fast Transient Surges (VFTS) in transmission lines and transformers [4]. Without mitigation, the switching transients originating from the circuit-breaker operation could also lead to the insulation breakdown in the windings and affect the transformers reliability [5]. If the spectrums of the transient surge voltages match with the transformer windings, resonant oscillations could be generated in the transformers. A high-frequency resonant oscillation is one of the causes for deterioration of insulation in transformer windings [2]. In addition, the surge voltages are threat to the insulation systems of the high voltage apparatus. The transient voltage resonances that are induced to the terminal of the windings could be superimposed on the input voltage waves of transformers. Generally, the external surges are unique in term of magnitude, rise time and delay time [6].

Transformer winding equivalent circuits are traditionally used to examine the voltage surge distribution in the transformer windings [7–9]. Several studies have been carried out to examine the transient voltage surge propagations through simulations and experimental setups [10–16]. Lumped equivalent circuit model have been widely used to analyze the resonant responses of windings subjected to switching surges [6, 17–20]. Various methods have been used to examine the transient voltage of transformer windings such as travelling wave theory, winding terminal characteristics and modal analysis [6, 21, 22]. The resonant responses of windings subjected to external overvoltage surges mainly depend on transformer windings geometrical design. The transformer’s terminal, inter-turn and inter-layer insulations may experience severe voltage stresses due to the amplitude and steepness of the external impulse. These voltage stresses if not mitigated could lead to failures of transformers and result in interruption of the supply to the users [1, 23]. Therefore, to ensure uninterrupted power delivery, it is important to appropriately protect the transformers against the possible internal or external voltage surges [24].

The non-linearity of the transient voltages can be mitigated through the modifications in design of the insulation in the transformer’s winding as per described in the IEEE PC62.69a/D3 [25]. Other common approach to mitigate the stated issues is the placement of the electrostatic shield as per recommended by IEC 60076–3 and IEC 60076–4 at predetermined locations in the winding geometry. Several studies have shown that it could mitigate the effect of the transient voltages and improve the linearity of the voltage distributions [26–32]. In addition, it is shown that the placement of electrostatic shield is more economical as compared to other approaches [25]. In general, the thickness of the aluminium electrostatic shield can be determined based on the eddy current loss and physical geometry of the winding. The linear behaviour of the voltage distribution along the windings depends on the amplitude of the impulse whereby it is characterised by the factor, α [29, 30, 33, 34]. The value of α is dependent upon transformer’s geometrical structure. If α = 0, uniform distribution of voltage stresses is expected whereby the resonances along the winding will be low. As the value of α deviates from 0, the voltage stress distribution could be non-uniform, which lead to increment failures probability for thin insulation along the stressed conductors [35, 36]. The configuration of electrostatic shield placement depends on the location of the switching surges due to the dependency of the calculated RLC parameters on the mechanical construction of the winding structure. This paper presents an investigation on the effect of shield placement configurations on the transient voltages in a 33/11 kV transformer under Standard Switching Impulse (SSI) and Oscillating Switching Impulse (OSI) surges, which is the main contribution of this study. Other impulse types like standard lightning and chopped lightning impulses that exist in the power system have been considered in the earlier study [33]. Since the transient voltage impulses normally would be subjected to the outer surface of the windings, i.e., only the HV side of the transformer winding is applied with surge impulses [3, 36]. The resistance, inductance, and capacitance (RLC) elements of transformer winding are calculated, and the resulting transformer winding equivalent circuit is used for the transient voltage studies under SSI and OSI with different configuration of shield placements. The contribution of the study is the knowledge of the SSI and OSI impacts on the transient voltage surges in the transformer and evaluation of the effectiveness of shield as the chosen mitigation approach.

## Methodology

### A. The overall framework of the modeling of transformer winding

**Fig 1:**
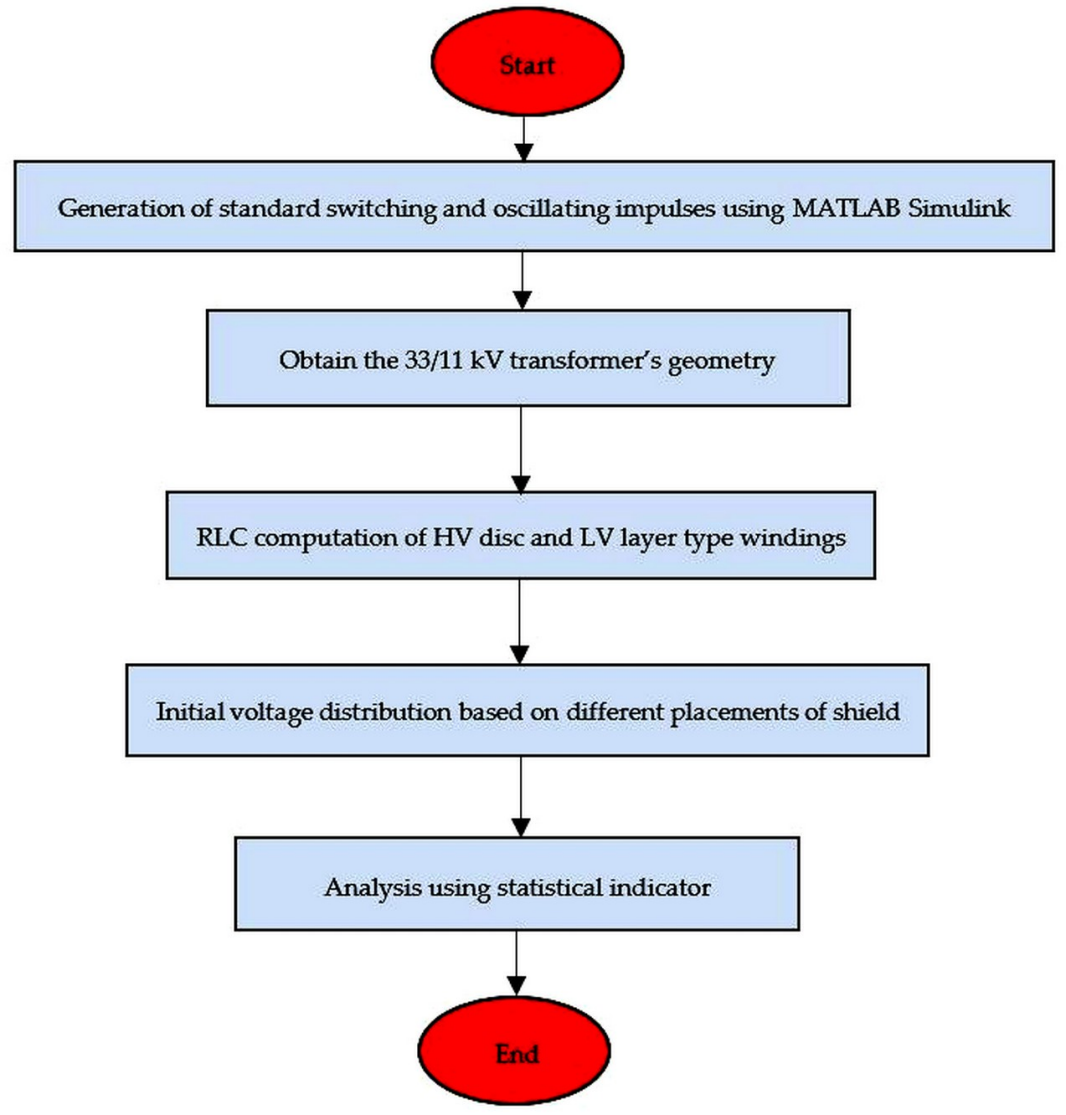
The framework to study the effect of the shield on the surge voltage distribution.

The overall framework of the switching studies in the 33/11 kV transformer can be seen in Fig 1. SSI and OSI were generated using MATLAB Simulink. The RLC parameters of both HV and LV windings of a transformer were computed based on its geometrical specifications. Next, the analysis was carried out for the different placements of the shield in the HV winding of the transformer based on MATLAB Simulink. The final step was to determine the effect of the shield on the surge distribution using statistical indicator.

### B. Generation of standard and oscillating switching impulse voltage

**Fig 2:**
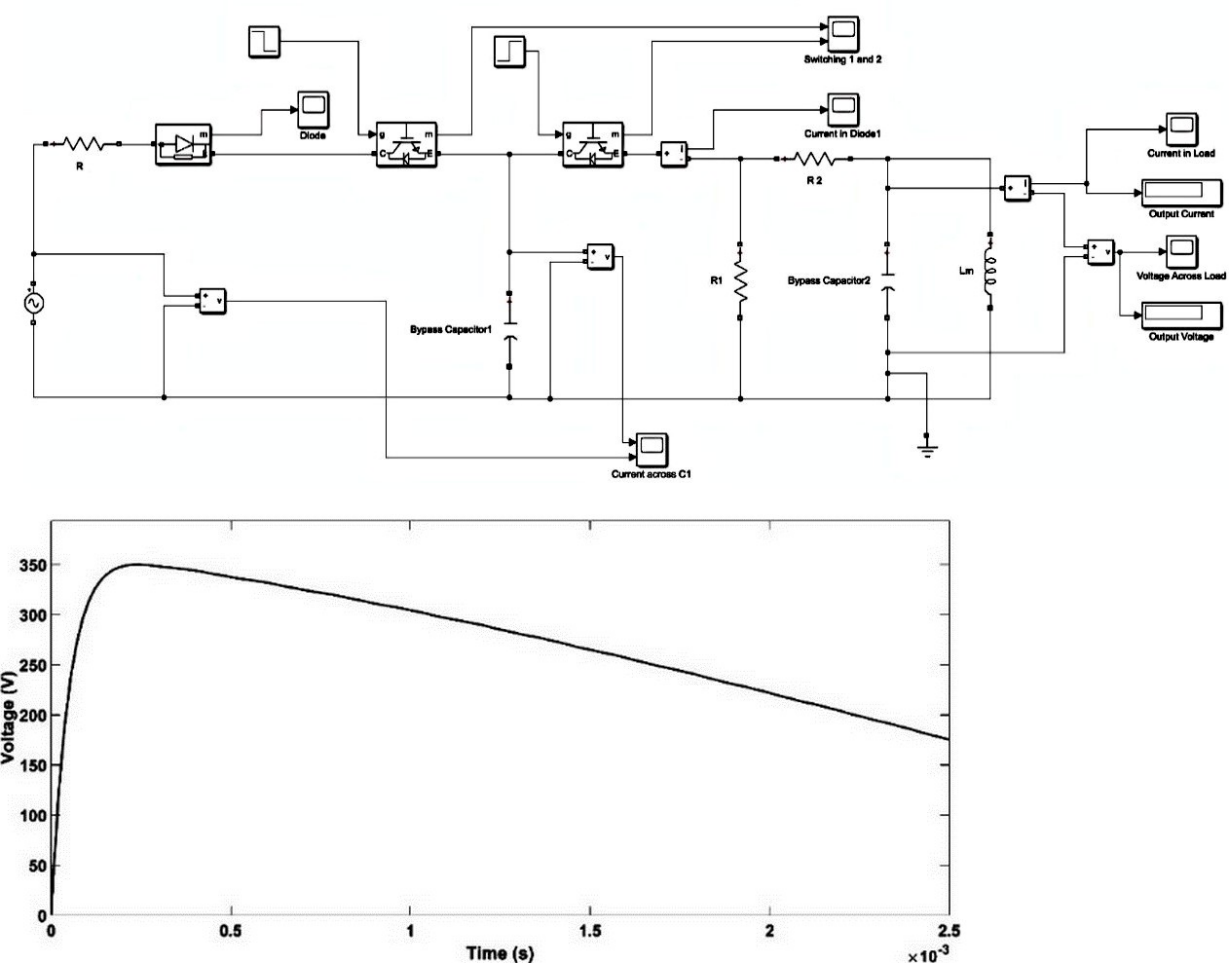
(A) Standard switching impulse generator circuit; (B) standard switching impulse voltage waveform (250/2500 μs).

A SSI of 250/2500 μs as per IEEE Std C57.98–2011 [37] was simulated based on the impulse generator Simulink circuit, as shown in Fig 2(A) and its parameters are shown in Table 1 [38]. The resultant SSI is shown in Fig 2(B).

**Table 1:** Impulse generator circuit specification for standard switching impulse.

Parameters	L	C_1_	C_2_	L_m_	R	R_1_	R_2_	U_c1_
**Units**	0	10 μF	1 μF	0.9 H	1000 Ω	1155 Ω	56 Ω	400 V

**Fig 3:**
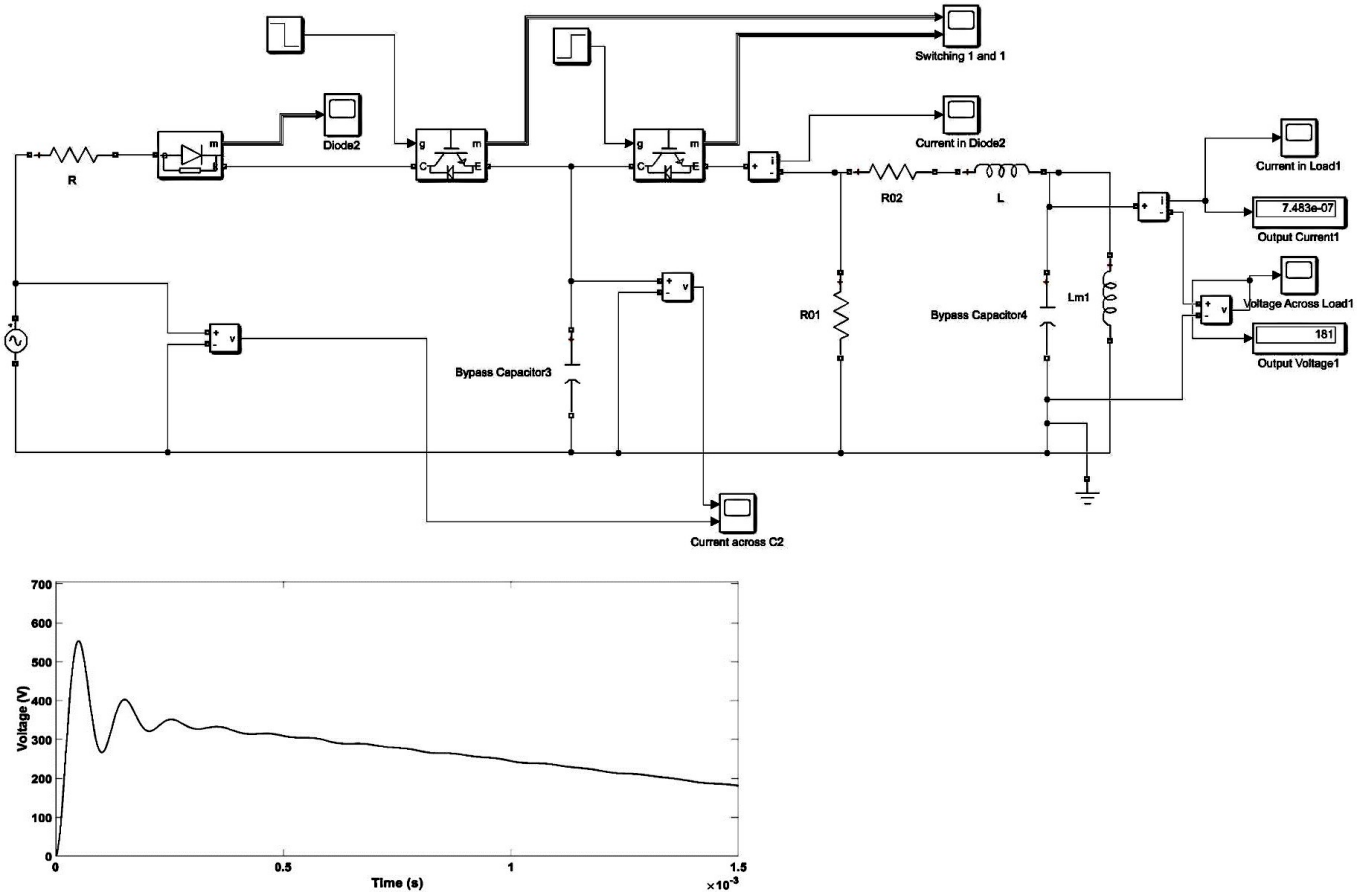
(A) Oscillating switching impulse generator circuit; (B) oscillating switching impulse waveform (50/1000 μs).

An oscillating switching impulse (OSI) waveform can be generated if the impulse generator circuit is underdamped (*R*_*2*_ < 2 [*L* (*C*_*1*_ + *C*_*2*_) / *C*_*1*_*C*_*2*_]-1) as seen in Fig 3(A). The only difference between SSI and OSI generator circuit is the absence of wave modulating inductance L, i.e., *L* = 0. An OSI was generated with peak time, *T*_*p*_ = 50 μs, half-peak time, *T*_*2*_ of 1000 μs and the amplitude of output voltage, *U*_*m*_ = 556 V and *f* = 10 kHz, which can be seen in Fig 3(B) and its parameters given in Table 2 [38]. The oscillations in the OSI waveform can be varied through variation of *L*. The frequency of OSI depends on *L*, *C*_*1*_, *C*_*2*_, and *R*_*2*_. The oscillatory frequency, *f* can be determined by Eq (1) and *T*_*p*_ in OSI is the oscillation period [38]:

$$f=\frac{1}{T_{P}}=\frac{1}{2\pi }\sqrt{\frac{C_{1}+C_{2}}{LC_{1}C_{2}}}$$ (1)

**Table 2:** Impulse generator circuit specification for oscillating switching impulse.

Parameters	L	C_1_	C_2_	L_m_	R	R_01_	R_02_	U_m_	T_p_	T_2_
**Units**	1.2 mH	3μF	0.22μF	1.3H	1000Ω	2230Ω	33Ω	556V	50μs	1000μs

### C. Calculation of RLC parameters

#### a. The construction of a disc-layer type 30 MVA, 33/11 kV transformer

**Fig 4:**
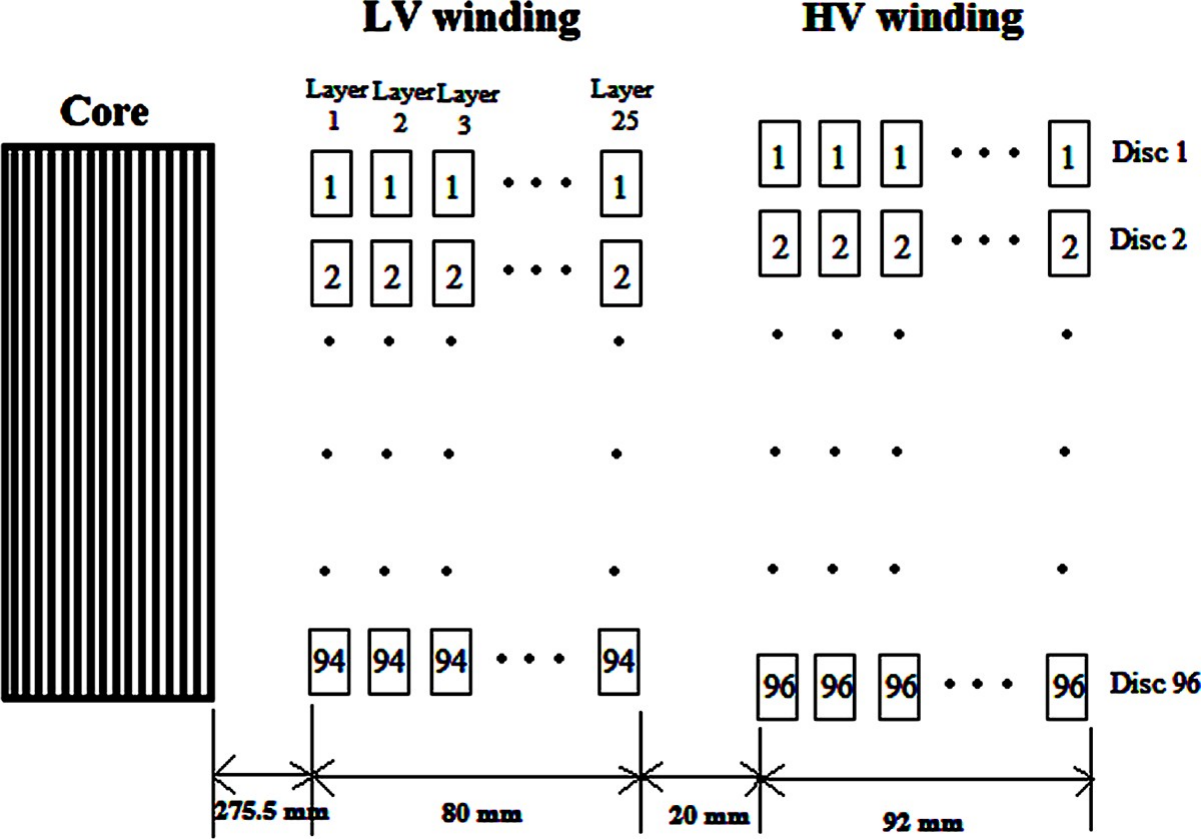
Front cross-sectional view of the winding for a single phase of the 33/11 kV transformer.

A Dyn11 transformer with the disc HV winding and layered helical LV winding with power and voltage ratings of 30 MVA, 33/11 kV was investigated as shown in Fig 4. The front cross-sectional view of the winding for a single-phase can be seen in Fig 4. The geometry specifications for HV and LV windings are shown in Tables 3 and 4.

**Table 3:** HV winding geometry specification of the 33/11 kV transformer.

Parameters	Values
Height of the conductor	11.5 mm
Width of the conductor	2.4 mm
Thickness of the insulation (double-sided)	0.5 mm
Number of turns per disc	30
Number of discs in one phase	96
Distance between each turn	3 mm
Cooling duct between turns 12 and 13	5 mm
Inner radius of the HV winding	374.5 mm
Outer radius of the HV winding	466.5 mm
Total circumference of the HV winding	79.17 m
Height of HV winding	1437 mm
Insulation between HV–LV windings (9 mm of Oil + 1 mm of pressboard)	20 mm
Relative permittivity of the insulation, *ε_r_*	2.3

**Table 4:** LV winding geometry specification of the 33/11 kV transformer.

Parameters	Values
Height of the conductor	11.5 mm
Width of the conductor	2.4 mm
Thickness of the insulation (double-sided)	0.5 mm
Number of turns per layer	94
Number of layers in one phase	25
Distance between each turn	3 mm
Cooling duct between layers 12 and 13	5 mm
Inner radius of the LV winding	275.5 mm
Thickness of the LV winding	80 mm
Outer radius of the LV winding	355.5 mm
Height of LV winding	1407 mm

#### b. Calculation of RLC parameters of disc type HV winding

The electrical parameters were calculated based on the height and width of the conductor, the number of turns in the disc and the thickness of the insulation and other details of the winding structure. The geometrical details of the HV winding are presented in Table 3.

The self and mutual inductances of the HV winding were calculated to form an inductance matrix of length 96 x 96 according to the equations in [39, 40]. The turn-to-turn capacitance, *C*_*t*_ between 2 adjacent conductors of a disc was calculated according to the equations in [39, 40]. The inter-disc capacitance, *C*_*d*_ between the adjacent discs was calculated based on equations in [39, 40]. Since the inter-turn capacitance, *C*_*t*_ between any two conductor is not the same due to the difference in conductor length of each turn, the total inter-turn capacitance, *C*_*t*,*total*_ was calculated and considered in series configuration. The total inter-disc capacitance, *C*_*d*,*total*_ was calculated and considered in parallel configuration. Since, the information on the grounded tank was limited for the transformer under study, the ground capacitance *C*_*g*_ was neglected. Consequently, the computation of α could not be carried out for the initial voltage distribution in the study. The resultant inter-disc capacitance *C*_*d*,*t*_ and the total series capacitance *C*_*s*_ of the whole winding as well as the total series capacitance of one disc were calculated according to [41]. The series resistance, *R*_*s*_ and conductance, *G*_*s*_ of the HV winding were calculated according to equations in [39, 40, 42] with consideration on the skin effect.

#### c. Calculation of RLC parameters of layered helical LV winding

The RLC parameters of the layered helical windings were calculated based on the geometrical specifications given in Table 4. The series inductances of each of the layers were obtained individually under the assumption that the corresponding layer as a single layer according to equations in [33, 43]. The mutual inductance, M between layers in the winding was computed based on equations in [39]. The resistance, *R*_*c*_ for the layered helical windings was calculated based on equations in [33]. The series capacitance between two adjacent turns, *C*_*tt*_ and the total series capacitance of layers of the whole winding, *C_tt_*′ as well as the capacitance between two layers, *C*_*ll*_ from layer 1 and layer *n* were determined according to equations in [33, 41, 42] at *r* = ∞.

The capacitances of the HV-LV intermediate barrier, *C*_*HV-LV*_ between HV and LV windings of the transformer and the permittivity, *ε_res_* of the gap which consists of oil impregnated insulation paper wrapped around the copper conductors were determined based on the equations in [34] with consideration on the two adjacent layers. The series capacitances of the winding layer and the equivalent layer capacitance of two layers, *C_ll_*′ was calculated based on equations in [33].

### D. A disc type 33/11 kV transformer model

**Fig 5:**
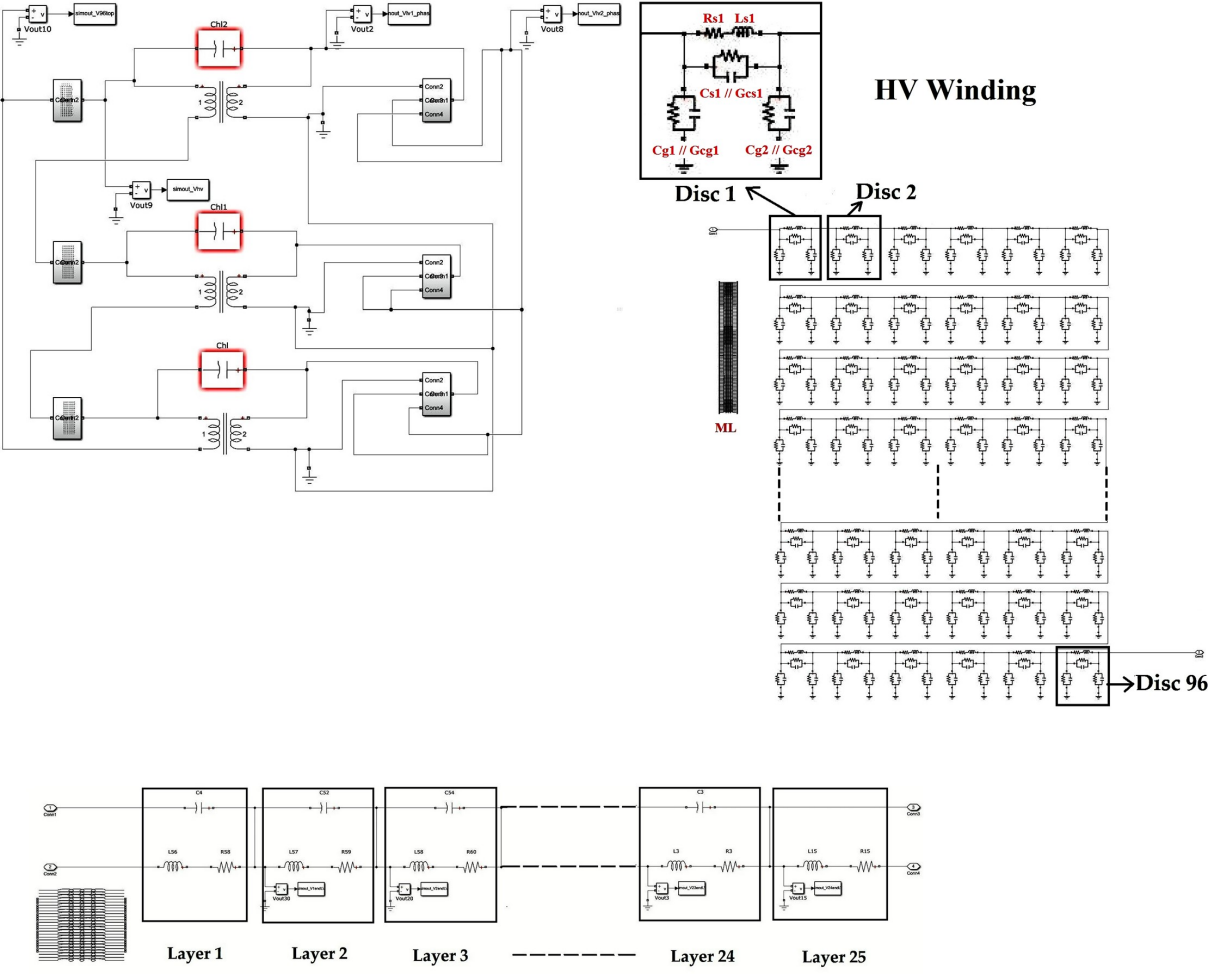
(A) Transformer equivalent circuit with 96 discs of HV layers in the winding and 25 layers of LV winding; (B) schematic diagram of 96 discs of the HV winding in the subsystem; (C) schematic diagram from layer 1 to layer 25 of LV winding in the subsystem for the 33/11 kV transformer.

A 33/11 kV Dyn11 transformer equivalent RLC circuit with its corresponding mutual inductances for both HV and LV windings can be seen in Fig 5A. The HV winding of a single phase consists of 96 discs and each of the discs has 6 conductors with 5 turns. The insulation thickness between each of the conductors is 0.5 mm. There is a cooling duct with 5 mm thickness between turns 12 and 13. The distance between each of the discs is 3 mm. The distance between HV and LV winding is 20 mm. The inner and outer radiuses of the HV winding are 374.5 mm and 462.5 mm respectively. Each of the sections is represented by a disc and considered in the lumped RLC parameters of the HV winding. The 96 discs HV winding inside the subsystem can be seen in Fig 5B and the 25 layers of LV winding inside the subsystem is shown in Fig 5C. The RLC values of HV and LV windings can be seen in Table 5 and S1 Table. *Rs* is the series resistance, *L*_*s*_ and *M*_*L*_ are the self and mutual inductances for each of the discs. *Rs* and *Ls* were connected in series. *C*_*s*_ and *G*_*cs*_ are the total series capacitance and conductance that are connected in parallel. *C*_*g*_ and *G*_*cg*_ are the ground capacitance and conductance between ground and winding conductor that are connected in parallel. In addition, the effects of transformer core were not considered since the magnetizing inductances of the core were not dominant in the current high frequency model [44, 45]. An assumption was made whereby the HV and LV windings were connected to represent an ideal transformer to simplify the calculation. The skin effect of the LV winding was not considered in the calculation since the focus of the study was on the HV winding behavior under overvoltage. In addition, the losses induced through the skin effect were found to be low in the LV winding [46]. The validation of the 3-phase transformer model had been carried out in [33] through the comparison between calculated and simulated values of the initial voltage distribution.

**Table 5:** RLC parameters of the HV winding for the 33/11 kV transformer.

Parameter	Values
Turn-to-turn capacitance, *C*_*t*_	33.5 pF
Total inter-disc capacitance, *C*_*d*_	526.89 μF
Series capacitance, *C*_*s*_	208.68 μF
Series Resistance, *R*_*s*_	4.67 Ω
Conductance *G*_*s*_	131.12 μΩ
Capacitance between HV and LV winding, *C*_*HV-LV*_	3494.95 nF

### E. Shield placement configurations

#### a. Case 1: Shield placement as conductor 7

**Fig 6:**
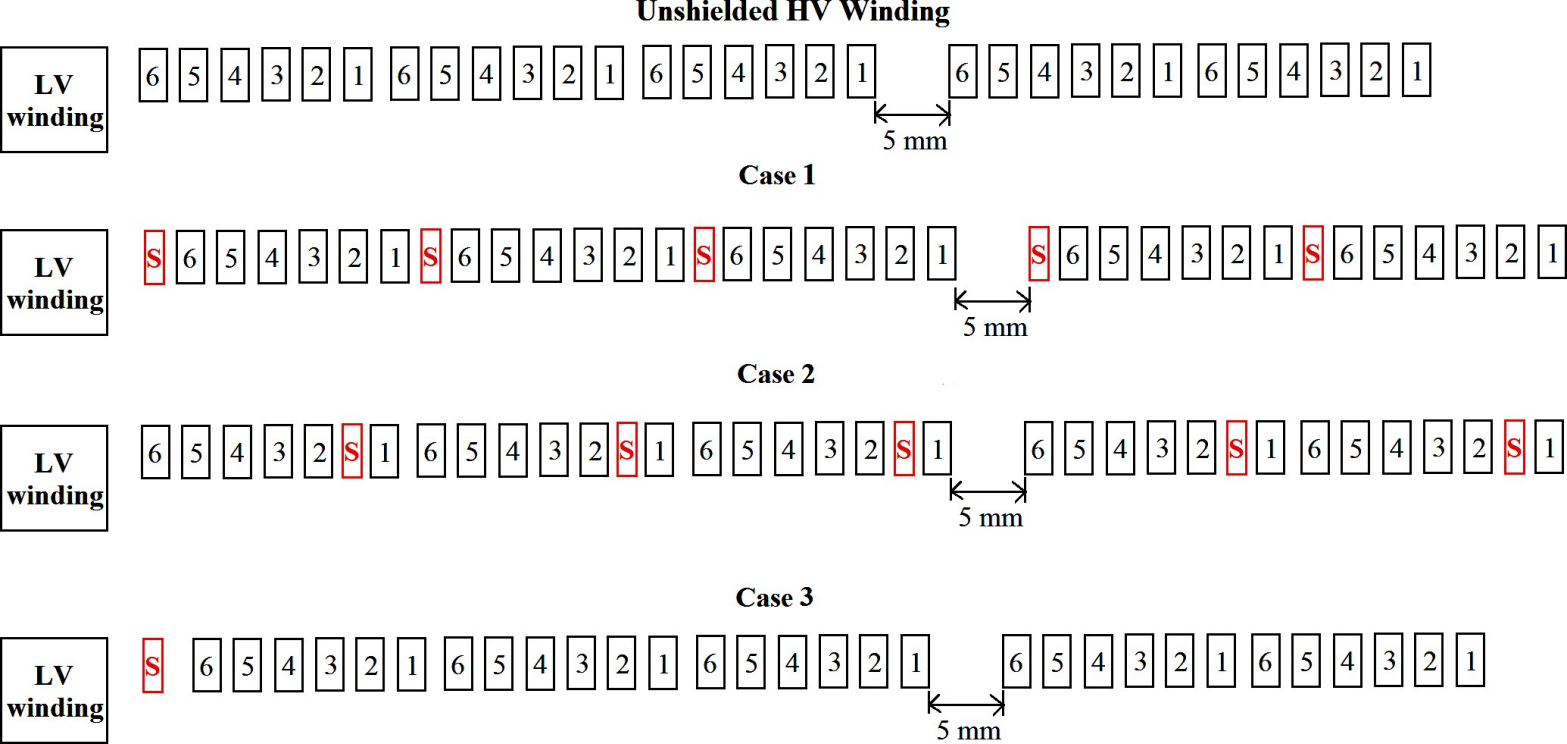
Transformer winding model with different cases of shield placement.

An electrostatic aluminium shield with thickness and length of 0.075 mm and 11.5 mm was placed as conductor 7 in the HV winding. Since the purpose of the shield is not for handling of current, the size should be kept as small as possible to preserve the compactness of the transformer winding and it should possesses low stray loss [47]. The shield with a thickness of 0.075 mm was used in the study due to low manufacturing cost and low eddy current loss [48, 49]. The shield has 5 turns and it was placed as floating potential as shown in Fig 6. Since there are 5 turns of the shields, the outer radius of the HV winding was increased 5 times to 0.375 mm. The introduction of the electrostatic shield increased the series capacitance. The winding model of the transformer and the updated RLC values can be seen in Fig 6 and Table 6.

**Table 6:** Existing and updated RLC parameters of HV winding with shield placement for the 33/11 kV transformer.

Parameter	Without shield	Shield after Conductor 7	Shield between Conductor 1–2	Shield between HV and LV windings
Turn-to-turn capacitance, *C*_*t*_	31.8 pF	33.5 pF	31.79 pF	31.77 pF
Total inter-disc capacitance, *C*_*d*_	526.89 μF	533.06 μF	527.11 μF	526.98 μF
Series capacitance, *C*_*s*_	208.68 μF	211.11 μF	208.77 μF	208.73 μF
Series Resistance, *R*_*s*_	4.67 Ω	4.68 Ω	4.68 Ω	4.68 Ω
Conductance *G*_*s*_	131.12 μΩ	132.65 μΩ	131.17 μΩ	131.11 μΩ
Capacitance between HV and LV winding, *C*_*HV-LV*_	3494.95 nF	3481.63 nF	3494.95 nF	3481.63 nF

#### b. Case 2: Shield placement between conductors 1 and 2

The shield was placed as floating potential between conductors 1 and 2 and it was considered as additional conductor as shown in Fig 6. The outer radius of the HV winding was increased by 0.375 mm. The RLC parameters for 5 inner turns of the conductor experience almost no change as compared to no shield condition as seen in Table 6.

#### c. Case 3: Shield placement between HV and LV windings

An electrostatic aluminium shield was placed between HV and LV winding. The shield consisted of 1 turn and it was placed as floating potential shown in Fig 6. The shield is helical in nature and it result in the displacement of HV windings by 0.075 mm outward. The updated model and the RLC values can be seen in Fig 6 and Table 6, respectively.

### F. Statistical indicator

The comparisons between the waveforms were performed by the statistical indices. Since the initial voltage distribution along the winding should be linear in nature, the standard errors of the slope (SEb) were employed to examine the effect of the shield to linearize the voltage distributions along the windings.

#### a. Standard error of the slope (SEb)

The standard error of the estimates (SEE) generates the magnitudes for the standard error of the slope (SEb) that can be used to assess computed and measured responses. If linear models are used for interpretation, SEE can be used to measure the deviation of the prediction points for the regression curve. i.e., the prediction point is $\hat{y_{it}}$ where the observed point is *y_it_* for magnitude i at the particular reference t. The average absolute difference between each of the predicted and observed points, i was defined as SEE. The behaviors of the transient voltage along the discs were analyzed to determine the dissimilarities in the slope. SEE and SEb were calculated based on Eqs (2) and (3) [50].

$$SEE=\sqrt{\sum \left(y_{t}-\hat{y_{t}}\right)}$$ (2)

$$SEb=SEE/{SD}_{d}\times \sqrt{n}$$ (3)

where *d* is the case of different slopes, *SD* is the standard deviation, and *n* is the number of signal points. The value of SEb should be 0 for a straight linear line.

The transient voltages of 11 discs from a total of 96 discs for the 33/11 kV transformer under switching surges were analyzed. The top-most disc was considered as 96 and discs 93, 83, 73, 63, 53, 43, 43, 33, 23, 13 and 3 were analyzed for the voltage distribution. The voltage impulse was applied to the top-most disc, 96 in the transformer model and the resultant transient voltage waveforms at the 3 upper discs 93, 83 and 73 were shown in the study. Only HV winding was considered in this study.

The eddy current loss was neglected for the transformer under study since the calculated values were found to be low as compared to the calculated total loss. The mutual coupling between HV and LV windings was also not considered since it was shown that the transformer turns ratio is equal to the ratio of inductance transferred [51].

## Results

### A. Transient voltage in the 33/11 kV transformer under SSI

#### a. Transient voltage under SSI without shield

**Fig 7:**
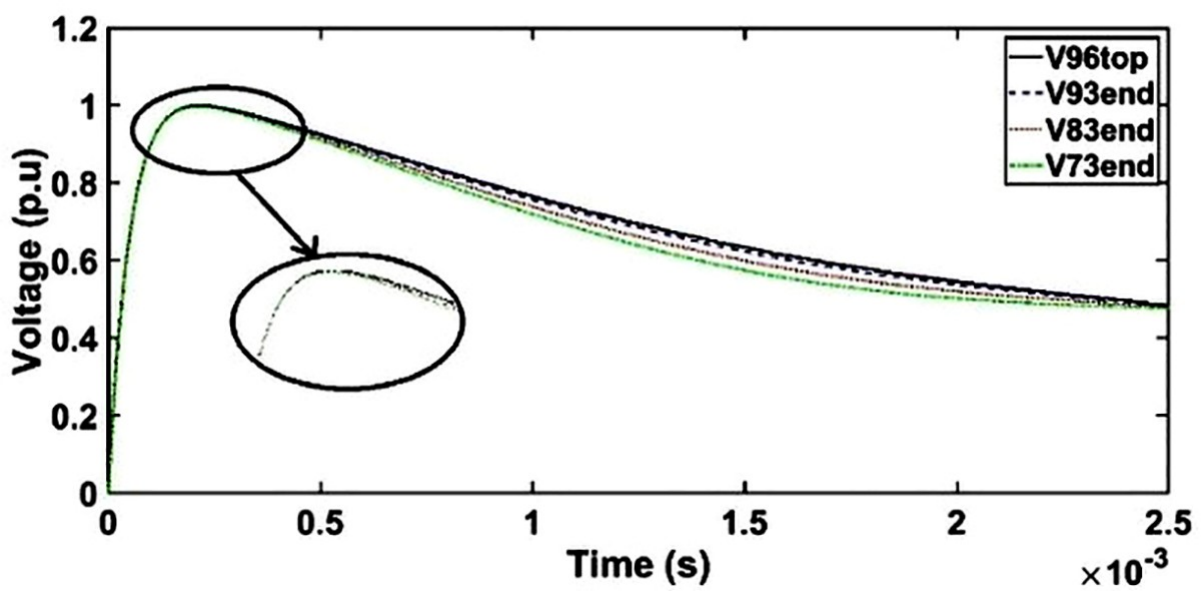
Transient voltage for unshielded winding under SSI.

The transient voltage under SSI for an unshielded HV winding can be seen in Fig 7. The voltage waveforms slightly deviate from the SSI at the wave tail region at time between 500 μs and 2400 μs.

#### b. Case 1: Transient voltage under SSI with shield placement as conductor 7

**Fig 8:**
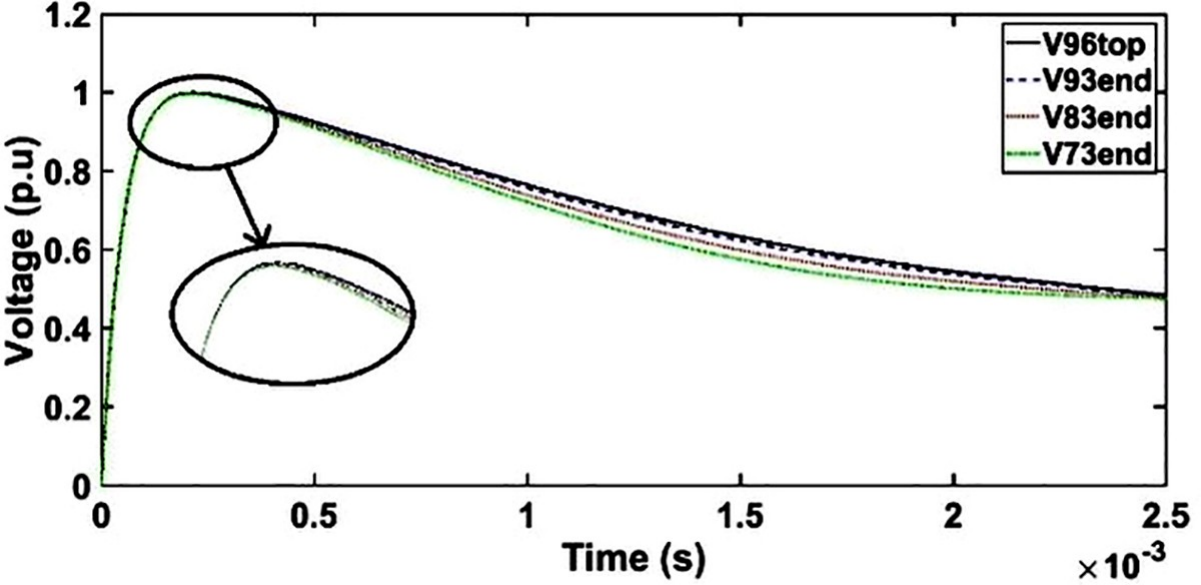
Transient voltage for shield placement as conductor 7 in HV winding under SSI.

Similar pattern as unshielded winding is observed for floating shield placement as conductor 7 in the HV winding whereby no resonance is observed and only slight deviation is found for transient voltage at the end of discs 93, 83 and 73 at time between 350 μs and 2450 μs as seen in Fig 8. The resonances are suppressed due to the decrement of the electromagnetic interferences among the winding conductors caused by the presence of electrostatic shield [52].

#### c. Case 2: Transient voltage under SSI with shield placement between conductor 1 and 2

**Fig 9:**
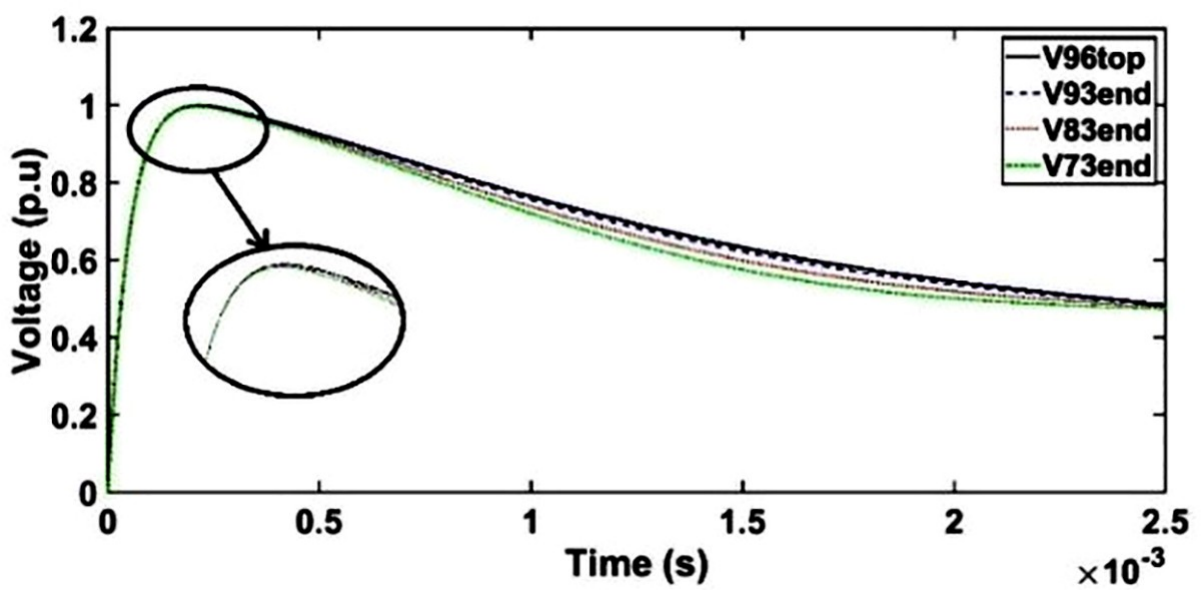
Transient voltage for shield placement between conductor 1 and 2 of HV winding under SSI.

The transient voltage in the transformer model with the placement of shield between conductors 1 and 2 can be seen in Fig 9. The voltage waveforms at the end of discs 93, 83 and 73 slightly deviate from SSI at time range between 400 μs and 2450 μs. Similar as unshielded winding, no resonant phenomenon is found.

#### d. Case 3: Transient voltage under SSI with shield placement between HV and LV windings

**Fig 10:**
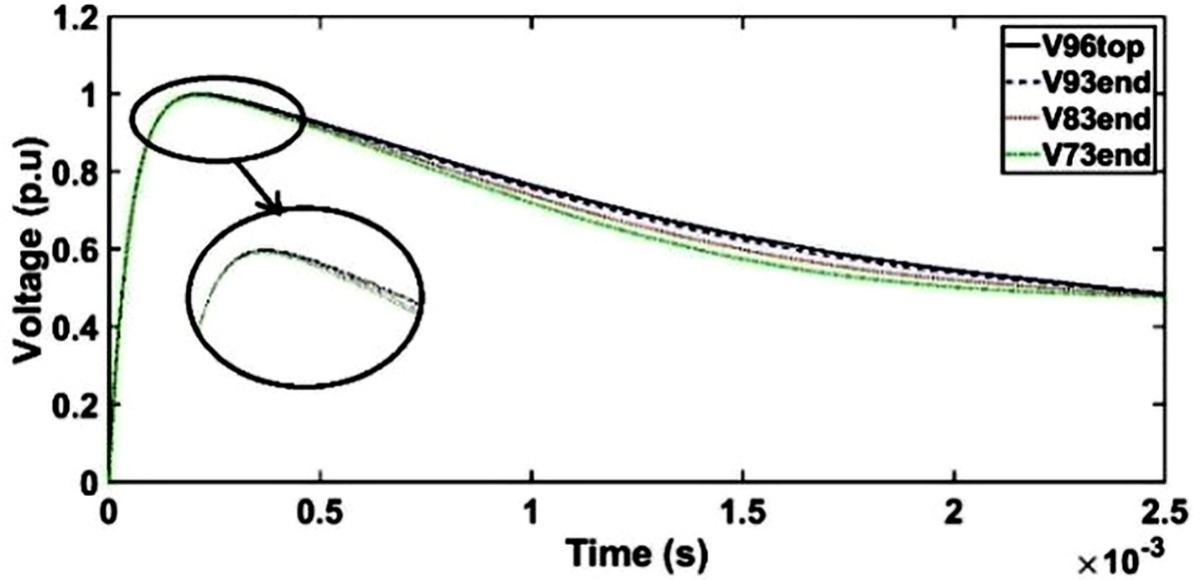
Transient voltage for shield placement between HV and LV windings under SSI.

The placement of shield between HV and LV windings leads to similar responses as unshielded winding as shown in Fig 10. The transient voltage at the end of discs 93, 83 and 73 slightly deviate from SSI at time between 350 μs and 2450 μs and no resonant is observed.

### B. Transient voltage in the 33/11 kV transformer under OSI

#### a. Transient voltage under OSI without shield

**Fig 11:**
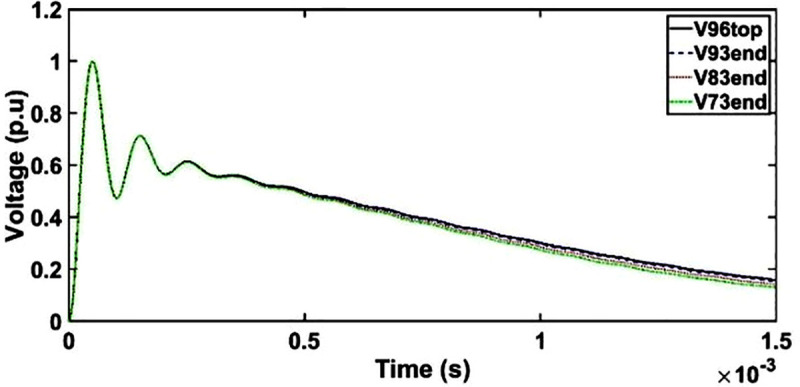
Transient voltage for unshielded winding under OSI.

The transient voltage at the end of discs 93, 83 and 73 slightly deviates from OSI at the wave tail region from 500 μs to 1500 μs as seen in Fig 11. However, the variations in the transient voltages are not observed at the end of discs 93, 83 and 73 as compared to disc 96 from 0 μs to 500 μs. It should be noted that the amplitude of the OSI depends on the type of an electrical equivalent circuit and time of the oscillating components in the transient voltage [16].

#### b. Case 1: Transient voltage under OSI with shield placement as conductor 7

**Fig 12:**
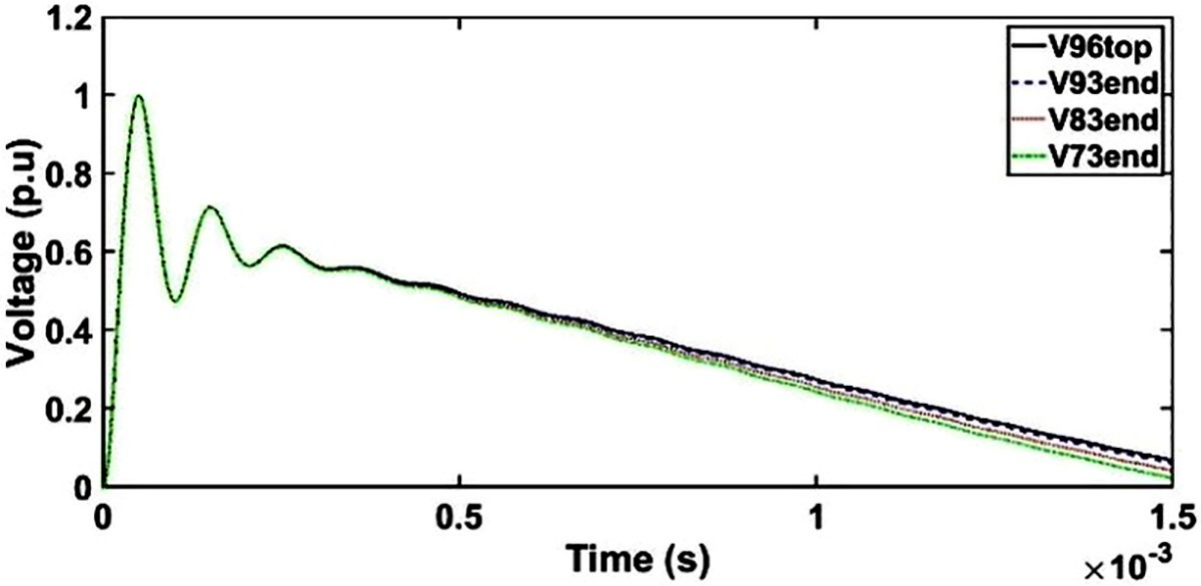
Transient voltage for shield placement as conductor 7 under OSI.

Similar as unshielded winding, the transient voltage at the end of discs 93, 83 and 73 for shield placement as conductor 7 deviates from OSI at the wave tail region from 600 μs to 1500 μs as seen in Fig 12.

#### c. Case 2: Transient voltage under OSI with shield placement between conductors 1 and 2

**Fig 13:**
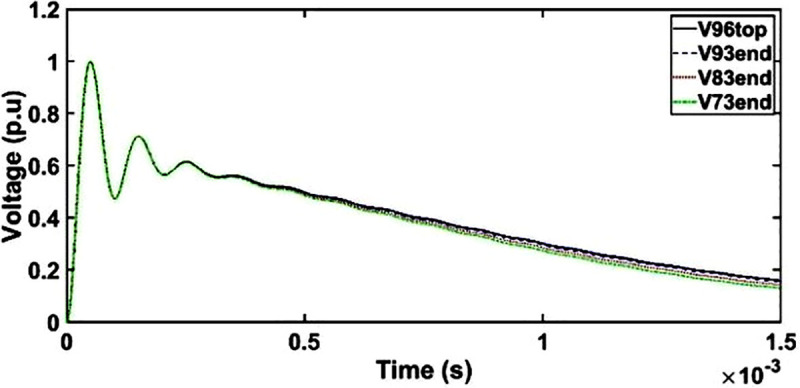
Transient voltage for shield placement between conductor 1 and 2 under OSI.

The transient voltage at the end of discs 93, 83 and 73 shows small deviation between 350 μs and 1500 μs as compared to OSI for shield placement between conductors 1 and 2 as seen in Fig 13.

#### d. Case 3: Transient voltage under OSI with shield placement between HV and LV windings

**Fig 14:**
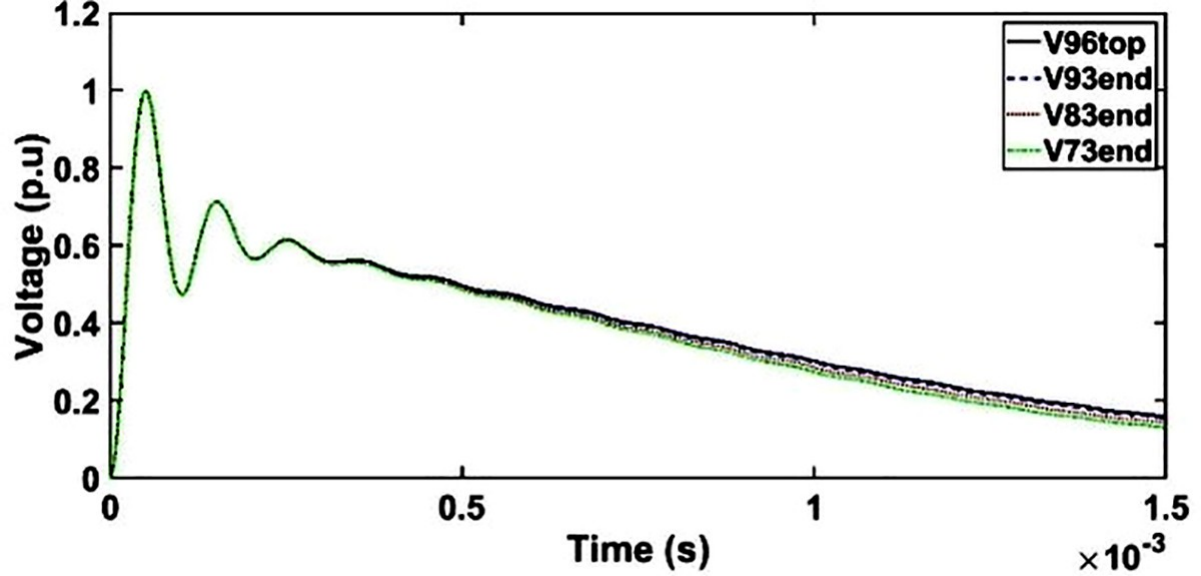
Transient voltage for shield placement between HV and LV windings under OSI.

The transient voltage at the end of discs 93, 83 and 73 for shield placement between HV and LV shows similar patterns as unshielded winding whereby only minor resonances between 300 μs and 1500 μs are observed as seen in Fig 14.

## Discussion on the effect of shield placement configuration on the transient voltage

The oscillatory nature of the transient voltages is due to the presence of RLC elements in the equivalent transformer winding circuit [33]. The voltage stresses generated in the winding discs or layers can be mitigated through the placement of aluminium electrostatic shield inside the winding geometry. This approach could increase the series capacitance between discs in disc type windings [28]. The constant, α would be adjusted close to 0 and subsequently improve the linearization of the voltage drop along the windings [26, 34]. The variation in transient voltage along the discs in the winding could initiate resonant oscillations that could enhance the voltage gradients and lead to the insulation failure between the conductors [32]. The magnitude of the external switching surges peak normally should be lower than the aperiodic voltages generated by the transformer. However, the external transient voltage impulse can excite the oscillations in the transformer winding even at a low magnitude, if the internal frequency of the voltage impulse matches the natural or resonance frequency of the transformer [19].

**Fig 15:**
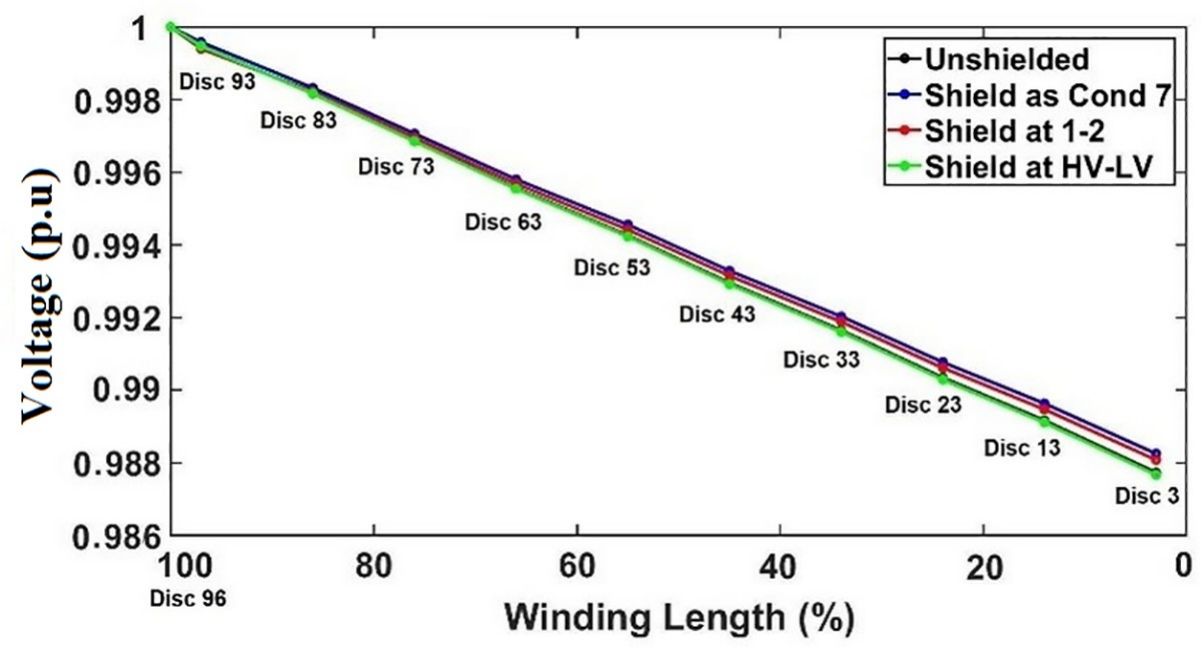
The effect of shield on initial voltage distribution under SSI.

**Fig 16:**
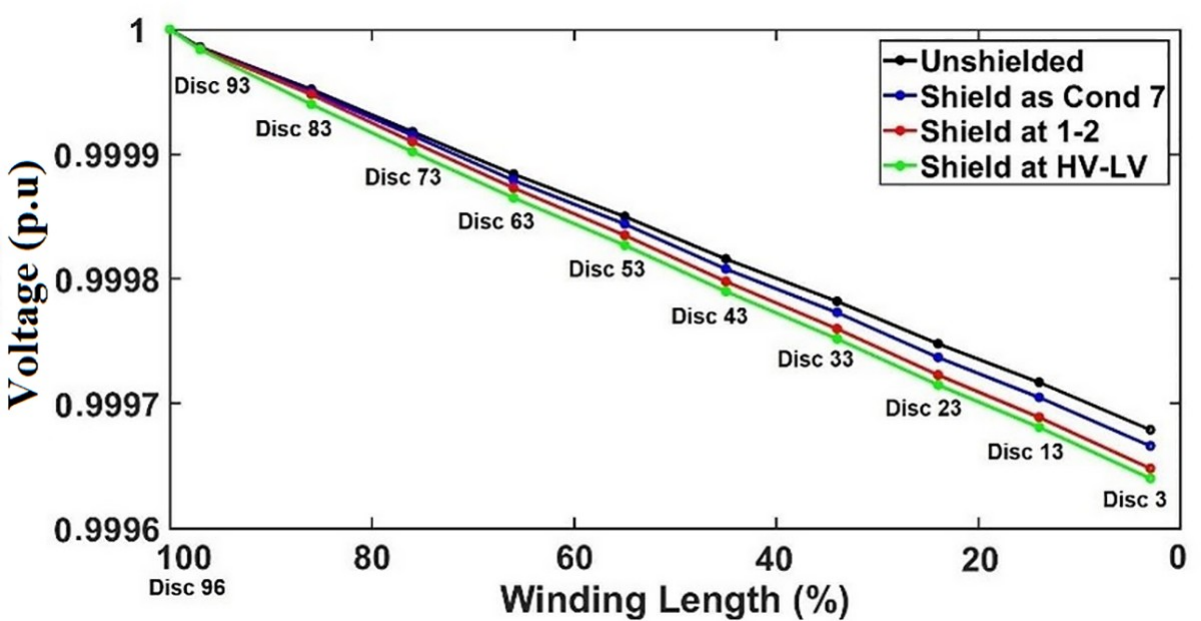
The effect of shield on initial voltage distribution under OSI.

The initial voltage distributions for each of the shield placements are obtained for transformer winding under SSI and OSI as seen in Figs 15 and 16. The SEb computed based on the initial voltage distribution for each of the shield placements under both SSI and OSI are listed in Table 7.

**Table 7:** SEb under SSI.

Shield placement	Slope Standard Error (SEb)	
	SSI × (10^12^)	OSI × (10^11^)
Unshielded	4.235	1.257
Shield as conductor 7	4.399	1.192
Shield between conductor 1 and 2	4.378	1.143
Shield at HV-LV	4.222	1.122

Under SSI, the voltage distribution curve linearization shows the optimize improvement as the shield is placed between HV and LV windings as shown in Fig 15. Placement of shield between conductors 1 and 2 significantly affects the linearization of the distribution of voltage whereby slight drop in voltage from disc 96 to disc 93. This phenomenon indicates a possibility of insulation breakdown between these discs due to the exertion of high voltage stress along the discs [53–56]. Placement of shield as conductor 7 results is similar voltage distribution curve linearization as unshielded winding which indicates that α is not equal to 0, since the curvature of the distribution curve for both the unshielded winding and the winding with shield placement as conductor 7 are higher than the linear behavior of the constant slope [33].

The shield placements as conductor 7 and between conductors 1 and 2 results in voltage drops along the discs which indicate α are not equal to 0 under OSI as shown in Fig 16. The voltage distribution curve linearization for shield placement between HV and LV windings is better than the unshielded winding whereby significant deviation is observed from disc 93 to disc 3.

The SEb values can be used to estimate the differences of the computed and ideal slope of initial voltage distribution curves. For the transformer windings under SSI, the placement of shield between HV and LV windings results in the lowest SEb with value of 4.222×10^12^ as seen in Table 7. Unshielded winding has lower SEb as compared to shield placement as conductor 7 and between conductors 1 and 2 as seen in Table 7.

Similar pattern as under SSI is observed for under OSI, whereby the lowest SEb is found with shield placement between HV and LV windings as shown in Table 7. However, shield placements as conductor 7 and between conductors 1 and 2 have lower SEb than the unshielded winding as seen in Table 7.

Based on the transformer under study, the placement of ungrounded shield between the HV and LV windings could decrease the resonant oscillations and results in linear distribution of voltages which subsequently could reduce the voltage stresses along the discs of the transformer winding.

## Conclusion

Based on simulation using RLC equivalent circuit of the transformer winding model, it is evident that the placement of shield between HV and LV winding results in linear distribution of voltages along the windings for the disc type transformer windings under SSI and OSI. In addition, SEb is the lowest with shield placement between HV and LV windings and has more linear voltage distribution under both SSI and OSI. Shield placements as conductor 7 and between conductors 1 and 2 do not improve the transient voltage along the discs of the transformer under SSI. However, these locations of shield placements improve the linearization of the voltage distribution curve under OSI.

Overall, it can be concluded that placement configuration is important to mitigate the transient voltage along the discs in order to ensure sustainable and uninterruptible power delivery via the transformers. Additional shields do not guarantee optimize transient voltage mitigation approach for the windings. This study could assist the manufacturers with a clear technical basis for the placement of shield for adequate protection for the transformer winding from the external transient switching voltage surges operating in the power system.

## Supporting information

S1 Table RLC parameters of the LV winding for the 33/11 kV transformer. (DOCX)
