# Supplementary material for: Effect of shield placement for transient voltage mitigation due to switching surges in a 33/11 kV transformer windings
Source: PLoS One. 2020 Oct 9;15(10):e0240368. doi: 10.1371/journal.pone.0240368 (PMC7546452; doi:10.1371/journal.pone.0240368)
Supplement: S1 Table — (DOCX) [file pone.0240368.s001.docx]

**S1 Table. RLC parameters of the LV winding for the 33/11 kV transformer.**

| **Layers** | **R (Ω)** | **L (µH)** | **C_ll_ (pF)** |
| --- | --- | --- | --- |
| 25 | 28.65 | 0.0025 | 4.77 e-6 |
| 24 | 28.13 | 0.0024 | 4.7701 e-6 |
| 23 | 28.18 | 0.0025 | 4.7701 e-6 |
| 22 | 27.94 | 0.0023 | 4.7702 e-6 |
| 21 | 27.71 | 0.0023 | 4.7703 e-6 |
| 20 | 27.47 | 0.0023 | 4.7704 e-6 |
| 19 | 27.24 | 0.0022 | 4.7704 e-6 |
| 18 | 27.01 | 0.0022 | 4.7705 e-6 |
| 17 | 26.77 | 0.0022 | 4.7706 e-6 |
| 16 | 26.54 | 0.0021 | 4.7707 e-6 |
| 15 | 26.30 | 0.0021 | 4.7708 e-6 |
| 14 | 26.07 | 0.0021 | 4.7708 e-6 |
| 13 | 25.83 | 0.0020 | 4.86 e-4 |
| 12 | 25.24 | 0.0019 | 4.77 e-6 |
| 11 | 25.00 | 0.0019 | 6.87 e-6 |
| 10 | 24.77 | 0.0019 | 4.7713 e-6 |
| 09 | 24.53 | 0.0018 | 4.7714 e-6 |
| 08 | 24.30 | 0.0018 | 4.7715 e-6 |
| 07 | 24.06 | 0.0018 | 4.7716 e-6 |
| 06 | 23.83 | 0.0017 | 4.7717 e-6 |
| 05 | 23.59 | 0.0017 | 4.7718 e-6 |
| 04 | 23.36 | 0.0017 | 4.7720 e-6 |
| 03 | 23.12 | 0.0016 | 4.7721 e-6 |
| 02 | 22.89 | 0.0016 | 4.7722 e-6 |
| 01 | 22.66 | 0.0016 | - |
